# Elevated Lactate Levels in Acute Pulmonary Embolism Are Associated with Prothrombotic Fibrin Clot Properties: Contribution of NETs Formation

**DOI:** 10.3390/jcm9040953

**Published:** 2020-03-30

**Authors:** Michał Ząbczyk, Joanna Natorska, Agnieszka Janion-Sadowska, Krzysztof P. Malinowski, Marianna Janion, Anetta Undas

**Affiliations:** 1 Institute of Cardiology, Jagiellonian University Medical College, 31-202 Krakow, Poland; michal.zabczyk@uj.edu.pl (M.Z.); j.natorska@szpitaljp2.krakow.pl (J.N.); 2 John Paul II Hospital, 31-202 Krakow, Poland; 3 The Faculty of Medicine and Health Sciences, The Jan Kochanowski University, 25-317 Kielce, Poland; ajanion@o2.pl (A.J.-S.); asianat@o2.pl (M.J.); 4 Institute of Public Health, Faculty of Health Sciences, Jagiellonian University Medical College, 31-126 Krakow, Poland; krzysztof.piotr.malinowski@gmail.com

**Keywords:** acute thrombosis, fibrinolysis, lactate, NETosis, pulmonary embolism

## Abstract

Background: Elevated plasma lactate levels correlate with high mortality rate in acute pulmonary embolism (PE) patients. We hypothesized that elevated lactate levels correlate with prothrombotic fibrin clot properties and enhanced neutrophil extracellular trap (NET) formation in acute PE. Methods: As many as 126 normotensive acute PE patients (aged 58 ± 14 years) were enrolled. Plasma fibrin clot permeability (K_s_), clot lysis time (CLT), endogenous thrombin potential (ETP), citrullinated histone H3 (citH3), and plasminogen activator inhibitor-1 antigen (PAI-1), together with plasma L-lactate levels were evaluated on admission. Results: Lactate levels ≥2 mM were found in 70 (55.6%) patients in whom we observed 29% higher neutrophil count and 45% elevated plasma citH3 levels. Elevated lactate levels were associated with more prothrombotic fibrin properties as reflected by 11% reduced K_s_, 13% longer CLT, along with 11% increased ETP. Lactate levels were positively associated with plasma citH3 concentrations, ETP, CLT, and PAI-1 (*p* < 0.05). An increase of lactate levels by 1 mM leading to the prolongation of CLT by 8.82 min was shown in the linear regression. Conclusions: Our findings suggest a new mechanism contributing to a negative impact of elevated lactate levels on prognosis in acute PE patients, in particular hypofibrinolysis, associated with enhanced NET formation.

## 1. Introduction

Acute pulmonary embolism (PE), isolated or combined with deep-vein thrombosis (DVT), is the major cause of mortality or hospitalization due to venous thromboembolism (VTE) [1]. Mortality related to PE has not been reduced for many years, mainly due to a short-term prognosis, and treatment strategies remain challenging [2]. Growing evidence indicates that right ventricular (RV) dysfunction or injury markers, namely increased brain natriuretic peptides (BNP) or troponin levels along with RV dysfunction, indicate worse prognoses [3,4]. The currently recommended markers to classify PE severity and the risk of early death have a suboptimal predictive value [4]. 

Lactate levels are considered as a laboratory parameter associated with disturbed oxygen supply in tissues [5]. Increased lactate levels have been also proposed as a prognostic marker in sepsis [6]. Vanni et al. have shown plasma lactate level ≥2 mM to be associated with higher mortality of acute PE patients, independent of shock, hypotension, RV dysfunction, or cardiac injury markers [7]. Moreover, elevated plasma lactate level has been considered as a predictor of short-term PE-related adverse events, suggesting that this parameter may be important as a decision-making factor during PE care [8].

L(+)-Lactate is a main stereo-isomer of lactate present in human blood. D(–)-lactate concentrations are low, about 1%–5% of those of L(+)-lactate. A previous in vitro study performed using bovine neutrophils showed that lactic acid is able to activate neutrophil extracellular trap (NET) release and promote neutrophil adhesion [9]. Moreover, lactic-mediated NET formation was dependent on peptidyl arginine deaminase 4 (PAD4) [9], a key enzyme in DNA decondensation and NETosis. Several studies have explored diverse mechanisms by which NETs can enhance thrombosis [10]. It has been shown that NETs activate platelets and provide a scaffold for red blood cells and procoagulant proteins, including von Willebrand factor (F), fibronectin, fibrinogen [10], FXII [11], and tissue factor (TF) [12], demonstrating the complex crosstalk between blood components and NET formation [13]. Fibrin clots enriched in histone–DNA complexes had higher stability, rigidity, and prolonged clot lysis time [14]. NETs have been shown to increase in vitro thrombin generation in septic patients’ blood [15]. As far as we know, there are no reports showing whether lactate concentrations in acute PE patients are linked to the prothrombotic changes in circulating blood.

Thus, we aimed to investigate if plasma lactate levels are correlated with NETosis-mediated plasma fibrin clot property alterations in acute PE patients.

## 2. Material and Methods

### 2.1. Patients

As many as 126 acute symptomatic PE white patients were recruited from December 2016 to March 2019 in four cardiology centers, all of whom were emergency admissions. This groups was described in detail previously [16]. Briefly, the diagnosis of PE was based on the presence of typical symptoms and positive results of high resolution spiral computed tomography. The following exclusion criteria were applied: diagnosed cancer, pregnancy, postpartum period, high-risk PE with shock or hypotension, ischemic stroke in the past 3 months, myocardial infarction in the past 3 months, end-stage kidney disease, vitamin K antagonist use, and anti-Xa activity ≥0.2 U/mL suggesting the effect of heparins.

A simplified PE severity index (sPESI) was assessed [4]. sPESI equal to 0 indicated low-risk PE. Normotensive PE patients with sPESI of ≥1 represented an intermediate-risk group. Using the ESC approach, patients with both RV dysfunction (by echocardiography or CT angiography) and elevated cardiac troponin T (TnT) represented an intermediate–high-risk category, while those with normal RV or TnT levels were classified as an intermediate–low-risk group. 

RV dysfunction was recognized in the presence of RV dilatation in the absence of significant left ventricular disease, based on the ESC guidelines [4]. DVT was diagnosed if thrombi were visualized in lower extremity deep veins using color duplex sonography performed within the first 48 h since enrolment. We categorized VTE episodes as those without any identifiable cause (unprovoked) or those associated with well-established factors (provoked) including known cancer, recent major surgery or trauma, plaster cast or hospitalization (within the preceding 28 days), or pregnancy or delivery in the past 3 months. Arterial hypertension was defined as elevated systolic and/or diastolic blood pressure ≥140/90 mmHg in consecutive measurements after clinical stabilization, or the current antihypertensive therapy. Coronary artery disease (CAD) was diagnosed as a history of documented myocardial infarction, hospitalization due to unstable angina, or prior elective percutaneous coronary intervention. Heart failure was diagnosed if the New York Heart Association (NYHA) class was 2 or higher or/and the left ventricular ejection fraction was less than 45%. The definition of diabetes was as follows: fasting plasma glucose ≥7.0 mmol/L (126 mg/dL) on two separate occasions or the current hypoglycemic treatment.

The study participants were followed by telephone contacts and clinic visits scheduled at 3 and 12 months since the index PE event. The primary composite endpoint was the occurrence of recurrent PE and/or DVT or death. The Jagiellonian University Ethical Committee approved the study, and participants provided informed written consent.

### 2.2. Laboratory Variables 

Before initiation of anticoagulant therapy on admission, blood samples were collected from an antecubital vein with minimal stasis. Blood cell count, glucose, creatinine, prothrombin time (international normalized ratio, INR), activated partial thromboplastin time (aPTT), lipid profile, D-dimer, N-terminal B-type natriuretic propeptide (NT-proBNP), and high-sensitivity TnT were assayed by routine laboratory techniques in the hospital laboratory. Positive high-sensitivity TnT was defined as a value ≥14 pg/mL and positive NT-proBNP as a value ≥600 pg/mL [4]. FVIII activity (Siemens Healthcare Diagnostics, Marburg, Germany) was measured by the Behring Coagulation System (Siemens). Anti-FXa activity was measured using a chromogenic assay (BIOPHEN, Hyphen-Biomed, Neuville-Sur-Oise, France). Fibrinogen was determined using the Clauss method. C-reactive protein (CRP) was measured by nephelometry (Siemens Healthcare Diagnostics). Plasma L(+)-lactate (Abcam, Cambridge, UK; sensitivity 0.02 mM, detection range 0.02–10 mM) levels were determined by an ELISA test. Based on the literature, abnormal plasma lactate concentrations were defined as values of 2 mM or greater [5,7]. P-selectin and platelet factor 4 (PF-4) (both Quantikine, R&D Systems, Inc., Minneapolis, MN, USA), as well as fibrinolysis inhibitors—plasminogen activator inhibitor-1 (PAI-1) antigen and thrombin activatable fibrinolysis inhibitor (TAFI) activity (both from Hyphen-Biomed, Neuville-Sur-Oise, France)—were assayed by ELISAs. Plasminogen and α2-antiplasmin (α2AP) levels were determined by chromogenic assays (Siemens). We also determined in plasma citrullinated histone H3 (citH3) (Cayman Chemical, Ann Arbor, MI, USA).

### 2.3. Fibrin Permeation Analysis

Fibrin clot permeation was determined as previously described [17]. Briefly, citrated plasma was mixed with 20 mM calcium chloride and 1 U/mL human thrombin (Merck KGaA, Darmstadt, Germany). Tubes containing the clots were connected to a reservoir of a Tris buffer, and we recorded the buffer volume percolating through the gels during one hour. A permeation coefficient (K_s_), Darcy’s constant, which is an indirect measure of the average pore size in the fiber network, was calculated from the equation: K_s_ = Q×L×η/t×A×Δp, where Q is the flow rate in time t; L is the length of a fibrin gel; η is the viscosity of liquid (in poise); A is the cross-sectional area (in cm^2^); and Δp is a differential pressure (in dyne/cm^2^).

### 2.4. Plasma Clot Lysis Assay

Fibrinolysis capacity was determined using a clot lysis time (CLT), as described in [18]. Briefly, 15 mM calcium chloride, 0.5 U/mL human thrombin (Merck), 15 µM phospholipid vesicles (Rossix, Mölndal, Sweden), and 20 ng/mL recombinant tPA (rtPA, Boehringer Ingelheim, Germany) were added to citrated plasma samples and then transferred to a microtiter plate. Absorbance was determined at 405 nm at 37 °C. We defined CLT as the time from the midpoint of the clear-to-maximum-turbid transition to the midpoint of the maximum-turbid-to-clear transition.

### 2.5. Endogenous Thrombin Potential

We used calibrated automated thrombography (CAT; Thrombinoscope BV, Maastricht, the Netherlands) to determine a key measure of thrombin generation in plasma samples, namely endogenous thrombin potential (ETP), the area under the curve, generated using the fluorometer (Ascent Reader, Thermolabsystems OY, Helsinki, Finland). 

Briefly, we added to platelet-poor plasma the reagent containing 5 pM recombinant TF, 4 µM phosphatidylserine/phosphatidylcholine/phosphatidylethanolamine vesicles, and FluCa solution (HEPES, pH 7.35, 100 nM CaCl_2_, 60 mg/mL bovine albumin, and 2.5 mM Z-Gly-Gly-Arg-7-amino-4-methylcoumarin). Each plasma sample was analyzed at 37 °C.

### 2.6. Statistics 

Variables are presented as numbers and percentages, mean ± standard deviation (SD) or median and interquartile range (IQR), as appropriate. Normality was assessed by the Shapiro–Wilk test. Equality of variances was assessed using Levene’s test. Differences between groups were compared using the Student or the Welch t-test depending on the equality of variances for normally distributed variables. The Mann–Whitney U-test was used for non-normally distributed variables. Categorical variables were compared by the Pearson chi-squared test or Fisher’s exact test. Multiple group comparisons were performed using analysis of variance (ANOVA) or the Kruskal–Wallis test. The Tukey–Kramer HSD test or Steel–Dwass method was used for the post-hoc comparisons. Univariate and multivariate logistic and linear regression models were performed to identify determinants of prolonged CLT. The multivariate model was fitted using backward stepwise regression. Variables that were associated with the prolonged CLT with a significance level of *p* < 0.2 in the bivariate models were selected for possible inclusion in the multivariate logistic regression models. All statistical analysis were performed using STATISTICA software Version 12.5 (StatSoft STATISTICA™, Krakow, Poland).

## 3. Results

We studied 126 patients aged 58.2 ± 14.4 years (Table 1). Positive TnT was found in 42 (33.3%), and positive NT-proBNP in 47 (37.3%) patients, while RV dysfunction was observed in 46 individuals (36.5%).

### 3.1. On Admission

**Figure 1:**
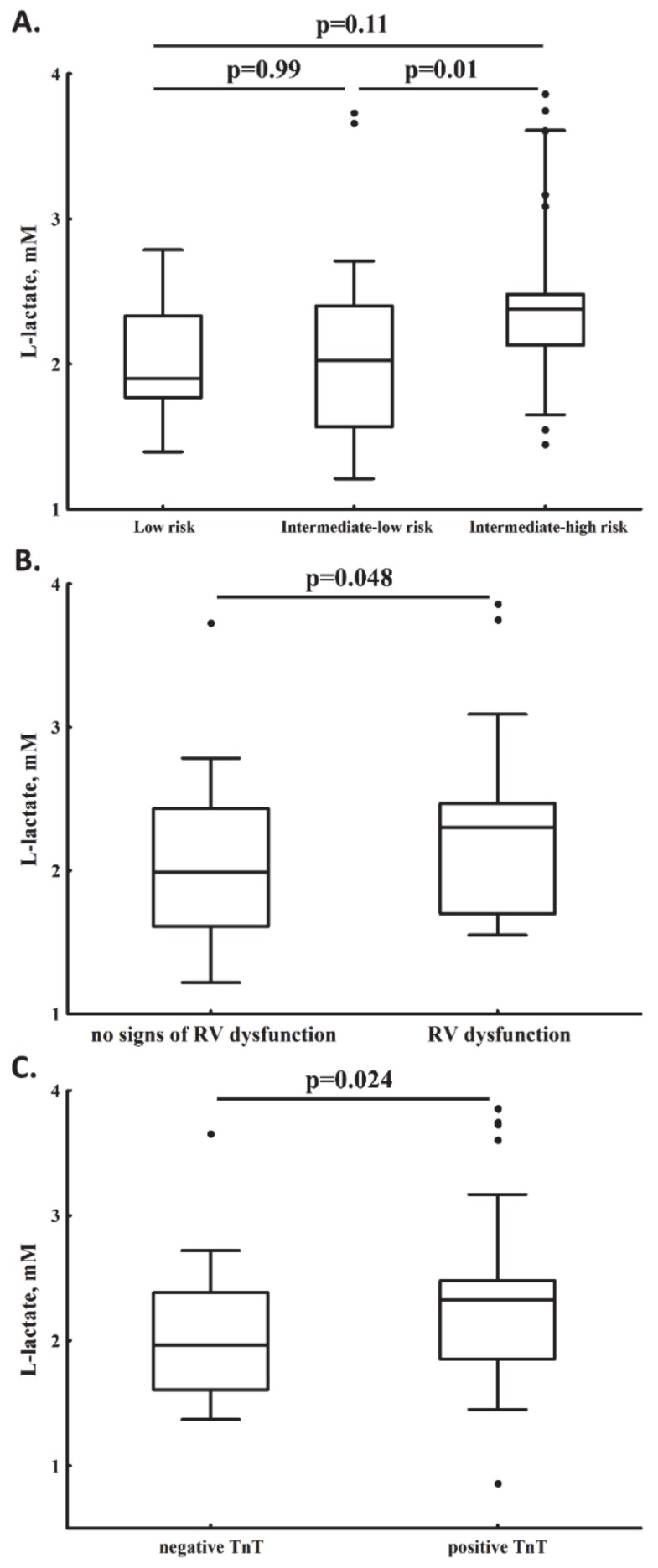
Plasma lactate levels determined in acute pulmonary embolism patients with low, intermediate–low, and intermediate–high early mortality risk (panel **A**, *p* < 0.05 for ANOVA) as well as in subjects with or without right ventricular (RV) dysfunction (panel **B**), and in those with positive or negative troponin T (TnT; panel **C**).

As many as 70 (55.6%) patients had lactate levels ≥2 mM. Patients with elevated lactate levels were older and more often had a history of CAD compared to the remainder (Table 1). PE patients with intermediate–high compared to intermediate–low mortality risk had 17.2% higher plasma lactate levels (2.38 (2.13–2.48) vs. 2.03 (1.57–2.40) mM; Figure 1A). RV dysfunction was associated with elevated plasma lactate levels by 15.6% as compared to the remainder (2.30 (1.70–2.47) vs. 1.99 (1.61–2.43) mM; Figure 1B), while subjects positive for TnT had 17.8% higher lactate levels than those with normal TnT (2.32 (1.85–2.4) vs. 1.97 (1.61–2.38) mM; Figure 1C).

Patients with plasma lactate levels ≥2 mM were characterized by 36.9% higher white blood cell (WBC) and 29% higher neutrophil counts along with 44.7% elevated citH3 levels (Table 1). Lactate concentrations were associated with WBC count (r = 0.18, *p* = 0.043), but not neutrophil count and CRP. Moreover, patients stratified according to lactate levels ≥2 mM compared with the remainder had higher plasminogen activity (+8.7%) and increased ETP (+10.9%; Table 2). Elevated lactate levels were associated with more prothrombotic fibrin properties as reflected by 10.9% reduced K_s_ and 12.6% longer CLT (Table 2), also adjusted for age and fibrinogen. 

Plasma lactate levels were positively associated with citH3 (r = 0.33, *p* = 0.0002), PAI-1 antigen (r = 0.28, *p* = 0.0018), ETP (r = 0.25, *p* = 0.0047), and CLT (r = 0.25, *p* = 0.0052), but not K_s_. Multivariate logistic regression revealed that after exclusion of factors strongly associated with CLT such as PAI-1, plasma lactate levels along with intermediate–high PE risk, BMI, and hsCRP were the independent predictors of prolonged CLT in acute PE patients (Table 3). The linear regression analysis showed that an increase of lactate levels by 1 mM led to the prolongation of CLT by almost 9 min (estimate = 8.82, 95% confidence interval 2.69–14.97 per 1 mM increase). No such impact was noted for K_s_ in acute PE.

### 3.2. Follow-up

**Figure 2:**
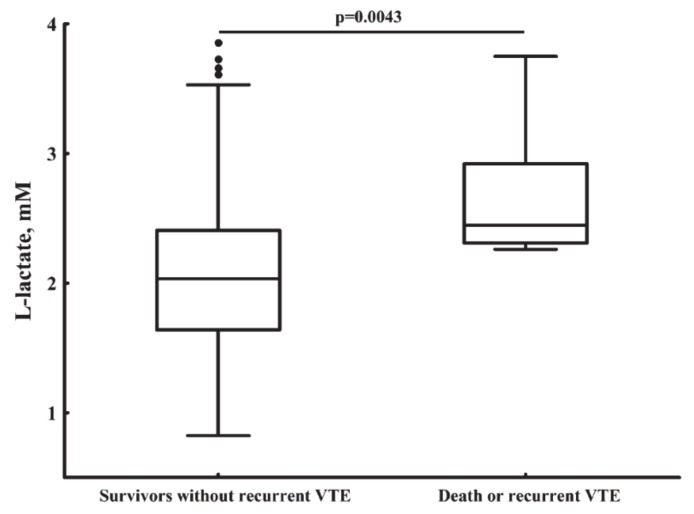
Plasma lactate levels assessed on admission in survivors without recurrent venous thromboembolism (VTE) vs. patients who died due to acute pulmonary embolism or its complications or experienced recurrent VTE during 1 year follow-up.

In total, we recorded nine (7.14%) deaths during a 1-year follow-up, including two patients with fatal DVT combined with PE, four patients with fatal stroke, one subject with sudden cardiac death, one with major bleeding followed by fatal acute myocardial infarction, and one trauma-related death. Distal DVT episodes were recorded in two (1.6%) subjects during follow-up. Patients who died or experienced recurrent VTE after acute PE compared to the remainder had 20.7% higher lactate levels on admission (2.45 (2.31–2.71) vs. 2.03 (1.63–2.41) mM, *p* = 0.0043; Figure 2) along with 50.5% higher citH3 levels (4.11 (3.66–5.62) vs. 2.73 (1.89–3.87) ng/mL, *p* = 0.00077). 

## 4. Discussion

We have shown for the first time that lactate levels ≥2 mM in acute PE patients are associated with impaired plasma fibrinolytic capacity accompanied by enhanced NET formation and increased thrombin generation. The current study suggests that hyperlactatemia contributed to unfavorably altered fibrin clot characteristics recently reported by us in acute PE [16]. In line with previous studies [3,7,8], we observed that lactate levels ≥2 mM are related to RV dysfunction and positive TnT, and they can predict intermediate–high mortality risk in acute PE. This study suggests the role of oxidative fibrinogen modifications, which may be caused by hypoxia in the presence of RV dysfunction. Previous reports have shown that there are many post-translational modifications of coagulation and fibrinolytic proteins, including fibrinogen molecule alterations associated with acute pathological conditions, such as inflammation or ischemia [19]. Moreover, our previous clot proteomics studies showed that beyond fibrinogen, many clot-bound proteins can determine its properties, for example platelet-derived factors or histidine-rich glycoprotein [20,21]. Biochemical reactions modifying fibrinogen molecules involve phosphorylation, hydroxylation, sulfation, oxidation, or nitration [19]. Such posttranslational fibrinogen modifications are associated with altered fibrinogen structure and/or function leading to prothrombotic fibrin clot phenotypes, including reduced clot porosity and susceptibility to lysis [22]. We found no differences in fibrinogen levels between PE patients characterized by normal or elevated plasma lactate levels. Heffron et al. [23] showed that fibrin polymerization correlates with fibrinogen nitration. We also did not observe differences in PAI-1 levels, known as a major determinant of CLT [24], in patients stratified according to plasma lactate levels, which suggest other factors involved in hypofibrinolysis in acute PE.

Monocytes and neutrophils, which are the major leukocytes in the inflammatory influx as well as within the venous thrombi generate nitrating metabolic products are able to induce fibrinogen nitration [25]. The myeloperoxidase (MPO), a major protein present in neutrophils, is suspected for promoting nitration [26]. Of note, MPO is a well-established marker of neutrophil activation and NET release. NETosis can be induced in a NADPH oxidase (NOX)-dependent and -independent manner [27]. The NOX-dependent pathway is activated by factors like pro-inflammatory interleukin 1β, tumor necrosis factor α cytokines, nitric oxide, or oxidized low-density lipoproteins [28,29,30]. NETosis is an energy-consuming process, and glycolysis in the source of energy during NET formation [27]. Glycolysis generates pyruvate, which under anaerobic conditions is converted to lactate, an end product of glycolysis. Lactate is a waste product of pyruvate metabolism; however, elevated lactate was shown to be associated with shock, sepsis, seizure, cardiac arrest, ischemia, and malignancy [31]. Increased lactate accumulation correlated with NET formation, while NET generation was inhibited by lactate dehydrogenase deactivation [27]. Therefore, we assumed that elevated lactate levels in acute PE patients might be considered as an early prognostic marker associated with NET formation, which in the acute thrombotic state can enhance coagulation activation. It should be highlighted that increased lactate levels occur due to enhanced stimulation of energy catabolism. However, coexistence of the inflammatory response can trigger impairment of mitochondrial function leading to metabolic acidosis [32]. We observed almost 45% increased citH3 levels in acute PE patients with plasma lactate concentrations ≥2 mM on admission. The presence of citH3 has been demonstrated within human thrombi, and citH3-positive cells were predominant in organizing thrombi obtained surgically or from autopsy, suggesting that attenuated NETosis may improve thrombolysis [33]. Moreover, extracellular histones, including H3, have been shown to enhance plasma thrombin generation in vitro [34]. Our data suggests that NET formation in association with increased thrombin generation contributes to slower fibrinolysis a few days since the acute PE symptom onset. A similar observation linking NET formation with fibrinolysis was shown in a recent report on type 2 diabetes [35]. Interestingly, the association of NET-related impairment of plasma fibrinolytic capacity in acute PE patients seems to be independent from fibrinogen or TAFI levels. As shown by Longstaff et al. [14], clots rich in DNA and histones are more resistant to lysis, and this is reversible by DNase. Recently, Mauracher et al. [36] showed that cancer patients with elevated citH3 levels at enrollment experienced more VTE episodes at 2 years than those with lower levels. The relevance of NETosis in acute PE diagnosed in noncancer patients underscores our observation that elevated circulating citH3 in acute PE is linked to higher early mortality risk. This hypothesis-generating study might support the concept that administration of DNase I could provide more effective thrombolytic therapy in acute patients with intermediate high-risk PE [10]. Our study may also suggest that due to a positive charge of NETs, unfractionated heparin (UFH) could be a potentially more effective anticoagulant in acute PE. It might be speculated that UFH could be preferred in acute PE patients in whom there is elevated lactate concentration ≥2 mM given a higher probability of suppressing intense NETosis. However, it has been recently shown that in vitro heparin can stimulate NETs formation, while the enoxaparin has a much lower ability to induce NETs than heparin, and fondaparinux does not induce NET formation [37]. The hypothesis on the potential benefits of heparin therapy in the context of real-life PE patients needs to be validated in a separate study.

The limitations of the current study are as follow: First, the studied group was relatively small. Second, high-risk PE patients, cancer and pregnant patients were excluded; therefore, our findings could not be extrapolated to these subsets. Cancer and pregnancy are known prothrombotic factors, which negatively alter fibrin clot phenotype [38,39] and modulate NET formation [39,40]. Third, all laboratory parameters were assessed on admission, while hyperlactatemia may be persistent [41] and might modulate inflammatory response and coagulation activation.

In conclusion, increased lactate levels correlated with increased NET formation as well as prothrombotic fibrin clot features, in particular impaired plasma fibrinolytic potential in acute PE patients assessed on admission. This study suggests a new mechanism leading to elevated lactate levels and prothrombotic clot phenotype, which translates to observed associations between elevated lactate levels and increased thrombin generation or enhanced NET formation. The practical implications of the current hypothesis-generating study need to be evaluated in a large group of patients.

## Figures and Tables

**Table 1:** Characteristics of patients with acute pulmonary embolism (PE) stratified according to serum lactate levels.

Variable	Acute PE Patients, n = 126	Lactate ≥2 mM n = 70 (55.6%)	Lactate <2 mM n = 56 (44.4%)	*p*-Value
Age, years	58.2 ± 14.4	60.9 ± 13.3	54.9 ± 15.2	0.02
Sex (male), n (%)	66 (52.4)	39 (55.7)	27 (48.2)	0.40
Body-mass index, kg/m^2^	28.1 ± 5.1	27.6 ± 4.6	28.8 ± 5.6	0.20
Current smokers, n (%)	25 (19.8)	15 (21.4)	10 (17.9)	0.62
**Comorbidities and medications**				
Time from PE symptom onset, days	4 (2–7)	3 (1–7)	5 (2.5–7)	0.08
Low risk PE, n (%)	20 (15.9%)	8 (11.4)	12 (21.4)	0.13
Intermediate–low risk PE, n (%)	77 (61.1%)	39 (55.7)	38 (67.9)	0.17
Intermediate–high risk PE, n (%)	29 (22.2%)	23 (32.9)	6 (10.7)	0.0033
First ever PE, n (%)	117 (92.9)	66 (94.3)	51 (91.1)	0.49
Concomitant DVT, n (%)	71 (56.3)	43 (61.4)	28 (50)	0.20
Coronary heart disease, n (%)	50 (39.7)	34 (48.6)	16 (28.6)	0.02
Hypertension, n (%)	68 (54)	38 (54.3)	30 (53.6)	0.94
Heart failure, n (%)	25 (19.8)	18 (25.7)	7 (12.5)	0.07
Diabetes mellitus, n (%)	42 (33.3)	28 (40)	14 (25)	0.08
Aspirin use, n (%)	40 (31.7)	26 (37.1)	14 (25)	0.15
Statins use, n (%)	77 (61.1)	44 (62.9)	33 (58.9)	0.65
**Laboratory investigations**				
White blood cell count, 10^3^/µL	7.03 (5.50–9.18)	8.20 (6.40–10.35)	5.99 (5.02–7.50)	<0.0001
Neutrophil count, 10^3^/µL	3.82 (3.10–5.77)	4.14 (3.55–6.69)	3.21 (2.59–4.06)	<0.0001
Hemoglobin, g/dL	13.8 ± 1.6	13.9 ± 1.5	13.6 ± 1.6	0.29
Platelet count, 10^3^/µL	220 (191–283)	221 (198–293)	218 (188–266)	0.65
Fibrinogen, g/L	3.26 (2.76–3.88)	3.35 (2.78–4.10)	3.17 (2.73–3.62)	0.09
High-sensitivity CRP, mg/L	3.65 (1.70–12.50)	3.51 (1.78–14.91)	4.20 (1.77–9.38)	0.65
D-dimer, ng/mL	3233 (1661–5325)	3102 (1622–5293)	3233 (1742–5642)	0.80
NT-proBNP, pg/mL	399 (106–1045)	491 (135–1261)	253 (92–742)	0.07
High-sensitivity troponin T, pg/mL	7.3 (1–51.4)	12 (6–78.4)	6.5 (1–40)	0.16
Citrullinated histone H3, ng/mL	2.77 (1.90–3.98)	3.40 (2.03–4.31)	2.35 (1.88–3.34)	0.010

Data are shown as numbers (%), mean ± standard deviation or median (1st quartile–3rd quartile). Abbreviations: CRP, C-reactive protein; DVT, deep vein thrombosis; NT-proBNP, N-terminal B-type natriuretic propeptide, PE, pulmonary embolism.

**Table 2:** Platelet markers, fibrinolytic proteins, thrombin generation, and fibrin clot parameters in patients with acute pulmonary embolism (PE) stratified according to serum lactate levels.

Variable		Acute PE Patients, n = 126	Lactate ≥2 mM n = 70 (55.6%)	Lactate <2 mM n = 56 (44.4%)	*p*-Value
P-selectin, ng/mL	77.1 ± 22.8		77.7 ± 23.3	76.5 ± 22.5	0.77
Platelet factor 4, ng/mL	69.4 ± 16.9		69.2 ± 17.3	69.7 ± 16.5	0.85
PAI-1, ng/mL	22.9 (16.7–33.2)		24.6 (17.3–35.4)	20.8 (15.8–30.3)	0.11
TAFI activity, %	100 (91–110)		100 (91–110)	100 (92–110)	0.63
α2-antiplasmin, %	104 ± 14		106 ± 16	102 ± 12	0.11
Plasminogen, %	108 ± 15		112 ± 16	103 ± 13	0.0013
ETP, nM × min	1660 (1494–1894)		1763 (1510–2096)	1590 (1460–1742)	0.013
K_s_, ×10^–9^ cm^2^	6.50 (5.46–7.40)		6.19 (5.20–7.10)	6.95 (5.95–7.50)	0.026
CLT, min	106.5 (95.0–121.6)		111.5 (98–128)	99.0 (88.5–113.5)	0.003

Data are shown as mean ± standard deviation or median (1st quartile–3rd quartile). Abbreviations: CLT, clot lysis time; ETP, endogenous thrombin potential; K_s_, fibrin clot permeability; PAI-1, plasminogen activator inhibitor type 1; TAFI, thrombin activatable fibrinolysis inhibitor.

**Table 3:** Determinants of prolonged CLT defined as values >121.5 min (top quartile) in patients with acute pulmonary embolism.

Variable	Univariate Analysis		Multivariate Analysis	
	OR (95% CI)	*p*	OR (95% CI)	*p*-Value
Age (per 1 year)	1.025 (0.995–1.056)	0.11	-	
Male sex	0.880 (0.394–1.964)	0.65	-	
BMI (per unit)	1.101 (1.015–1.193)	0.02	1.136 (1.032–1.252)	0.01
Intermediate–high PE risk	5.042 (0.056–12.363)	0.0004	3.197 (1.174–8.703)	0.023
RV dysfunction	2.540 (1.118–5.772)	0.026	-	
hsCRP (per unit)	1.022 (1.005–1.039)	0.93	1.020 (1.002–1.038)	0.031
L-lactate (per unit)	2.416 (1.238–4.713)	0.0097	3.074 (1.351–6.992)	0.007

Abbreviations: OR, odds ratio; CI, confidence interval; RV, right ventricular; for other abbreviations see Table 1 and Table 2.
